# PP16 Vial Sharing And Wastage Are Familiar Concepts, But What About Pack Sharing And Wastage?

**DOI:** 10.1017/S0266462324001892

**Published:** 2025-01-07

**Authors:** Sarah Kate Wilkes, Emily Eaton Turner, Neha Jiandani, David Thomson

## Abstract

**Introduction:**

The National Institute for Health and Care Excellence (NICE) conducts health technology assessment to assess cost-effectiveness and budget impact. For treatments provided in vials, NICE often considers how treatments are dispensed and adjusts the economic modeling costs accordingly. Vial sharing and wastage are likely familiar concepts to stakeholders, but the same consideration is not consistently given to tablet packs.

**Methods:**

Using anonymized examples, NICE assessed potential implications for cost-effectiveness and budget impact of different methods for modeling oral treatments. Firstly, the cost-effectiveness and budget impact were calculated based on a cost per milligram (mg) of treatment. The per mg cost was multiplied by the number of mg for each dose and did not account for the number of tablets or packs required. Using the same example, the cost per tablet was calculated by rounding each dose to the nearest whole tablet mg dose. Finally, the example was costed based on whole packs, which included the cost for any wasted tablets.

**Results:**

The anonymized examples showed that costing per mg versus per tablet versus per pack can have a significant impact on cost-effectiveness and budget impact. One example showed that treatment costs per 28 days could increase by over GBP1,000 (USD1,271) when costing per whole pack compared to per mg. This led to a difference in the incremental cost-effectiveness ratio (ICER) of nearly GBP10,000 (USD12,716). Another example demonstrated a potential increase in budget impact of nearly GBP1 million (USD1.27 million) per year. This magnitude of impact on cost-effectiveness and budget has the potential to change health technology assessment decisions and affordability in the United Kingdom.

**Conclusions:**

NICE is assessing an increasing number of oral treatments provided as tablet packs, not vials. This highlights the need to consider how pack sharing and wastage should be consistently considered in economic modeling. Developing standardized methods for modeling oral treatments would help ensure consistency of cost calculations and better reflect how treatments are dispensed in clinical practice.

